# RACIAL/ETHNIC DIFFERENCES IN IMPACTS OF COVID-19 ON PSYCHOLOGICAL WELL-BEING AMONG OLDER ADULTS

**DOI:** 10.1093/geroni/igad104.2670

**Published:** 2023-12-21

**Authors:** BoRin Kim, Esther Shin, Jihye Baek

**Affiliations:** University of New Hampshire, Durham, New Hampshire, United States; Illinois State University, Normal, Illinois, United States; Washington University in St. Louis, St. Louis, Missouri, United States

## Abstract

Covid-19 caused challenges to psychological well-being among older adults, but the impact may differ across various populations. The purpose of this study is to investigate racial/ethnic differences in psychological well-being among older adults during the first year of the pandemic, particularly focusing on mediating effects of different types of social support. Data came from the 2021 Health and Retirement Study (HRS), which includes a nationally representative sample of older Americans. Our sample was restricted to the respondents aged 65+ who participated in the 2021 HRS Perspectives on the Pandemic that focuses on the first-year pandemic experiences (N=4,656). We compared four racial/ethnic groups including white, black, Hispanic, and others. Psychological well-being was measured by the 12 questions on participants’ feeling during the first year of the pandemic (e.g., distressed, afraid, worried). Three distinct groups of help-receiving patterns were identified by latent class analysis: 1) no help, 2) intense help from kids, and 3) moderate help from diverse social relationships. Frequencies of in-person and not-in-person contacts with children or family were also included in our analysis. We found that Hispanic’s psychological well-being was the most negatively impacted by the pandemic (Coef.=0.687, p<.05) even after controlling for socio-demographic characteristics, physical and mental health. Our mediation analysis demonstrated that intense help from kids and less in-person contacts negatively mediated the association between Hispanics and their psychological well-being. This study shows that older Hispanics who had been known for strong family ties and support are at risk due to restricted social contacts during the pandemic.

